# The nonlinear impact of air pollutants and solar radiation exposure on the risk of hospitalisation for pterygium among adults in Shanghai, China: a time series analysis

**DOI:** 10.7189/jogh.15.04110

**Published:** 2025-04-11

**Authors:** Hongya Zeng, Yue Tan, Tong Lin, Lan Gong

**Affiliations:** 1 Department of Ophthalmology, Eye & ENT Hospital, Fudan University, Shanghai, PR China; 2 NHC Key laboratory of Myopia and Related Eye Diseases; Key Laboratory of Myopia and Related Eye Diseases, Chinese Academy of Medical Sciences, Shanghai, PR China; 3 Shanghai Key Laboratory of Visual Impairment and Restoration, Shanghai, PR China

## Abstract

**Background:**

In recent years, many studies have focused on the effects of air pollution on ocular surface health. However, there is currently little research on the relationship between pterygium and air pollution. We aimed to investigate the effects of air pollutants and solar radiation on the progression of pterygium in adults through a 5-year time series analysis.

**Methods:**

After collecting the meteorological data and clinical visits for pterygium in Shanghai, China from 2017 to 2023, we established a distributed lag nonlinear model (DLNM) for statistical analysis. We also conducted subgroup analysis according to age and sex to investigate the impact of risk factors on different populations.

**Results:**

This cohort included a total of 57 211 cases. We found that solar radiation, particulate matter less than 2.5 micrometre (μm) (PM_2.5_), ozone (O_3_) and nitrogen dioxide (NO_2_) all increased the risk of outpatient treatment of pterygium within a certain concentration range. Among them, PM_2.5_ and solar radiation have the most significant lag effects. The relative risk (RR) value was highest when the concentration of PM_2.5_ reach the peak value at a lag time of 13 days. Subgroup analysis showed that women and people aged 55 to 65 years were more susceptible to extremely high concentrations of PM_2.5_.

**Conclusions:**

Our results suggested that in addition to solar radiation, which is recognized as a risk factor for pterygium, PM_2.5_ exposure also seems to be related with an increase in the risk of pterygium. More targeted prevention and early interventions strategies remain to be studied.

Pterygium is a common chronic ocular surface disease, which mainly presents as epithelial proliferation and angiogenesis originating from abnormal limbal stem cells, often accompanied by inflammatory cell infiltration and the destruction of the Bowman’s membrane [1]. Although most cases are benign, severe pterygium can lead to vision impairment due to extensive corneal involvement in advanced stages [2]. In China, the estimated prevalence of pterygium in the general population aged 15–84 years is 9.84%, and shows an increasing trend with age. Due to the current demographics structure, the 55–59 age group contributed the largest number of patients, up to 11.78 million [3]. Therefore, to explore the risk factors for pterygium is of great public health significance.

Although the pathogenesis of pterygium has not been fully elucidated, the existing studies generally believe that its pathogenesis is closely related to the UV light in the environment. Due to the long-time exposure to the natural light, the ocular anterior segment can be easily injured by the UV rays in solar radiation, known as UV-associated eye diseases, mainly including pterygium, cataracts and acute UV keratitis [4,5]. Sunlight exposure as a risk factor for pterygium is now gaining attention, and ocular surface health monitoring for outdoor workers and the promotion of protective measures such as sunglasses have been proposed [6,7]. While UV exposure is a well-recognized risk factor, increasing urbanization and industrial development also highlight the need to examine the impacts of air pollutants on pterygium, as the ocular surface is exposed to both potential threats. Those air pollutants have shown positive correlation with dry eye, conjunctivitis, uveitis and other inflammatory ophthalmic diseases in many clinical studies, including particulate matter less than 2.5 micrometre (μm) (PM_2.5_), particulate matter less than 10μm (PM_10_), ozone (O_3_), carbon monoxide (CO), nitrogen dioxide (NO_2_) and sulphur dioxide (SO_2_) [8–11]. Although it also involves inflammatory changes in the cornea and conjunctivitis, the relationship between pterygium and air pollutants has not been fully reported, especially lacking in the studies combined with solar radiation and short-term time-lag analysis to alarm us of the risk factors of accelerated deterioration.

In this study, we obtained data on outpatient visits for pterygium from a major ophthalmic clinical centre in Shanghai, China over a five-year period, and planned to explore the relationship between the hospitalisation for pterygium and the short-term exposure to pollutants as well as solar radiation. As there is usually a time lag between the exposure to risk factors and the outpatient visits, the distributed lag nonlinear model (DLNM), which was proposed by Antonio and Gasparrini in 2010, was adopted for statistical analysis [11,12]. This model takes the lag time between the result and exposure as an additional correlation dimension, allowing the impact of a single exposure event to be distributed over a specific time period to account for the contribution of different lag time, and is especially suitable for quantifying the time process of the expose-response relationship in the studies about meteorological variables [13]. Since there is often a lag time between the patients' exposure to risk factors and their clinical visits, we used this model to quantify the nonlinear lag-effects of air pollutants and solar radiation on pterygium.

## METHODS

### Study region

Shanghai is one of the largest cities in China, being part of the alluvial plain of the Yangtze River Delta in the eastern frontier of China, and belongs to the subtropical monsoon climate zone. Located at about 120°52'–122°12' east longitude and 30°40'–31°53' north latitude, Shanghai consists of 16 districts with an administrative area of 6340.5 km^2^. By the end of 2023, the resident population of Shanghai is about 24.87 million. The spatial analysis of air pollutant concentration in China shows that the developed urban agglomerations in eastern China, represented by Shanghai, has long been the main distribution range of air pollutants related to urbanisation, such as NO_2_ and PM_2.5_, which has seriously threatened the health of residents [14,15]. Therefore, it is necessary to pay attention to the health effects of environmental stress in this region.

### Data collection

Our outpatient data collection was conducted from 1 January 2017 to 30 April 2023 at the Eye, Ear, Nose and Throat Hospital of Fudan University, which is considered to be one of the largest ophthalmic medical centres in Shanghai. We obtained the information of pterygium patients from the outpatient information system, including the date of visit, age, gender, and residence area under the authorisation of the ethics committees of the Eye, Ear, Nose, and Throat Hospital of Fudan University. According to the common accepted definition in the previous literature, pterygium was defined as a fibrovascular extension of the conjunctiva onto the clear cornea without any alternative explanation such as inflammation or trauma [16], and were diagnosed under a slit-lamp by experienced ophthalmologists based on the International Classification of Diseases, 10th Revision (ICD-10). Exclusion criteria was as follows:

1) patients under 18 years old;

2) patients whose residential area is not in Shanghai;

3) patients revisiting within 30 days;

4) without complete medical information.

The whole research was under the authorisation of the ethics committees of the Eye, Ear, Nose, and Throat Hospital of Fudan University.

Meteorological data mainly consists the daily average temperature (°C), daily average relative humidity (%) and daily average solar radiation intensity (watt per square metre, W/m^2^) in Shanghai from 2017 to 2023, provided by the Xihe Energy Meteorological Data Platform (https://xihe-energy.com/). Air pollutant data are collected from the fixed meteorological monitoring stations set by Shanghai Ecological Environment Bureau, including the 24-hour average concentrations of PM_2.5_ (microgram per cubic metre (μg/m^3^)), PM_10_ (μg/m^3^), SO_2_ (μg/m^3^), NO_2_ (μg/m^3^), CO (miligram per cubic metre (mg/m^3^)) and the eight-hour average concentrations of O_3_ (μg/m^3^) across the entire city area (https://sthj.sh.gov.cn/).

### Statistical analysis

First, a descriptive analysis of meteorological data, air pollutant concentrations and the number of outpatient visits in patients with pterygium were presented after eliminating incomplete data. The data characteristics are described by average value, standard deviation (SD) and the seasonal peak value of each meteorological variable. The possible correlation between various meteorological variables was examined using Spearman’s correlation.

The exposure-response relationship between the meteorological variables and outpatient visits was evaluated by a time-series model. Since the outpatient visits for pterygium typically follow a Poisson distribution, we used a Poisson generalised linear model as the basic framework. Finally, the model expression is as follows:

Y_t_ ~ Poisson(μ_t_)

𝐿𝑜𝑔(μ_t_) = 𝛼 + 𝑐𝑏(𝐴𝑖𝑟 𝑝𝑜𝑙𝑙𝑢𝑡𝑎𝑛𝑡𝑠_t_ , 𝑙𝑎𝑔) +cb (*Radiation_t_*, 𝑙𝑎𝑔) + 𝑛𝑠(𝐴𝑣𝑒𝑟𝑎𝑔𝑒 𝑡𝑒𝑚𝑝𝑒𝑟𝑎𝑡𝑢𝑟𝑒_t_ , 𝑑𝑓=3) + 𝑛𝑠( R𝑒𝑙𝑎𝑡𝑖𝑣𝑒 h𝑢𝑚𝑖𝑑𝑖𝑡𝑦_t_, 𝑑𝑓=3)+𝑛𝑠(𝑇𝑖𝑚𝑒_t_, 𝑑𝑓=7 × 14)) + 𝑎𝑠. 𝑓𝑎𝑐𝑡𝑜𝑟(𝐷𝑂𝑊_t_ ) + 𝑎𝑠. 𝑓𝑎𝑐𝑡𝑜𝑟(𝐻𝑜𝑙𝑖𝑑𝑎𝑦_t_ )

In this formula, t represents the date, while 𝑌_t_ refers to the daily counts of visits for pterygium and 𝛼 refers to the intercept. The matrix of the average daily concentration of air pollutants (𝐴𝑖𝑟 𝑝𝑜𝑙𝑙𝑢𝑡𝑎𝑛𝑡𝑠_t_) and the average daily intensity of solar radiation (*Radiation_t_*) is constructed by the cross-basis function (cb). Time_t_ represents the time trend, fitted by the natural cubic spline (ns) function to control the potential long-term and seasonal trends. As in our previous study, we defined the degrees of freedom (df) of temperature, humidity, solar radiation and air pollutants as 3, and the df of the natural cubic spline of time as 7 according to the Akaike information criterion (AIC) [11, 12]. The stability of the model was tested by adjusting the value of df [17]. Since the outpatient visits are also associated with holidays and weekends, we set up dummy variables to control for the confounding effects of the public holidays in China (𝐻𝑜𝑙𝑖𝑑𝑎𝑦_t_, 0 and 1 indicating non holidays and holidays) and days of the week (𝐷𝑂𝑊_t_). As we aim to explore the risk of progress in a short term, the maximum lag is set at 14 days.

To find out the populations that are more sensitive to meteorological factors, we evaluated the relationship between the outpatient visits and solar radiation as well as air pollutants in different subgroups. Considering the demographic structure, we conducted subgroups by gender (male, female) and age (18–54 years, 55–64 years, 65+ years) [3].

All data analysis and image in this study were performed in the *R*, version 4.2.3 (R Development Core Team, Vienna, Austria), which includes the 'dlnm', 'splines' and 'ggplot2' packages. A two-tailed *P*-value <0.05 was considered statistically significant.

## RESULTS

### Descriptive analysis

The descriptive analysis of daily outpatient visits for pterygium was conducted according to different subgroups (**Table 1**). Our cohort contained 57 211 cases altogether, with a daily mean of 24.77 and a SD of 17.32. In this cohort, 38 375 cases were female (67.1%) and 18 836 cases were male (32.9%). As for the different age groups, the largest number of cases were aged 55 to 64, accounting for 36.2% (n = 20 724), while 31.3% (n = 17 934) were under 55 and 32.4% (n = 18 553) were 65 or above.

**Table 1:** Descriptive summary of daily outpatient visits, 2017–2023

Variables	Summary	Average daily visits	SD
Total visits	57 211	24.77	17.32
Gender			
*Male*	18 836 (32.92%)	8.15	6.21
*Female*	38 375 (67.08%)	16.61	11.83
Age (year)			
*<55*	17 934 (31.35%)	7.76	5.78
*55 ~ 64*	20 724 (36.22%)	8.97	6.69
*> = 65*	18 553 (32.43%)	8.03	6.52

SD – standard deviation

**Figure 1:**
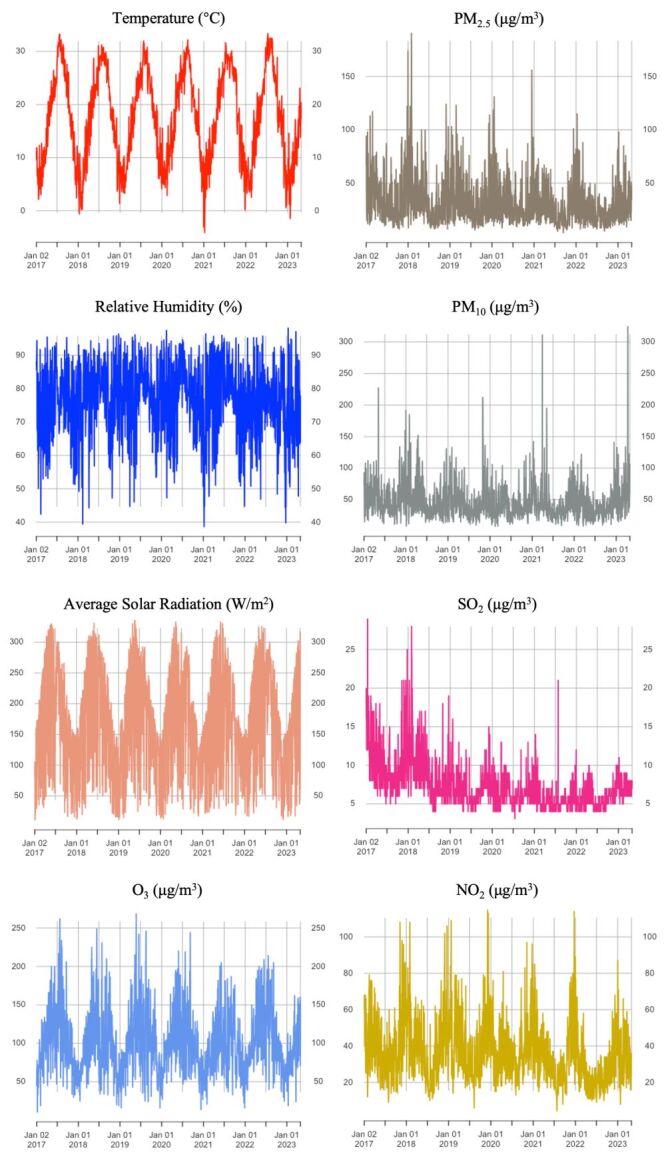
Daily changes meteorological factors and air pollutants in Shanghai, 2017–2023. CO – carbon monoxide, μm – micrometre, μg – microgram, NO_2_ – nitrogen dioxide, O_3_ – ozone, PM_2.5_ – particulate matter less than 2.5 μm, PM_10_ – particulate matter less than 10 μm, SO_2_ – sulphur dioxide.

The summary of meteorological variables showed some seasonal and periodic changes (**Figure 1**; Table S1 in the **Online Supplementary Document**). Among them, the daily average temperature and solar radiation intensity mainly increased in warm seasons (from April to September), while the concentration of air pollutants mainly increased in cold seasons (from October to March of the next year).

Using Spearman correlation analysis, we further evaluated the relationship between climate variables and air pollution (Table S2 in the **Online Supplementary Document**). The results showed that the concentration of PM_2.5_ was positively correlated with PM_10_, SO_2_, NO_2_ and CO (*P* < 0.01), and the strongest correlation was with CO (r = 0.80). In addition, there is a significant correlation between solar radiation and O_3_ (r = 0.67, *P* < 0.01). As PM_2.5_ is strongly positively correlated with CO and PM_10_ (r >0.70), we include only PM_2.5_ in the final model to avoid potential problems associated with multicollinearity referring to previous studies [18].

### Lag-response analysis between meteorological variables and pterygium visits

**Figure 2:**
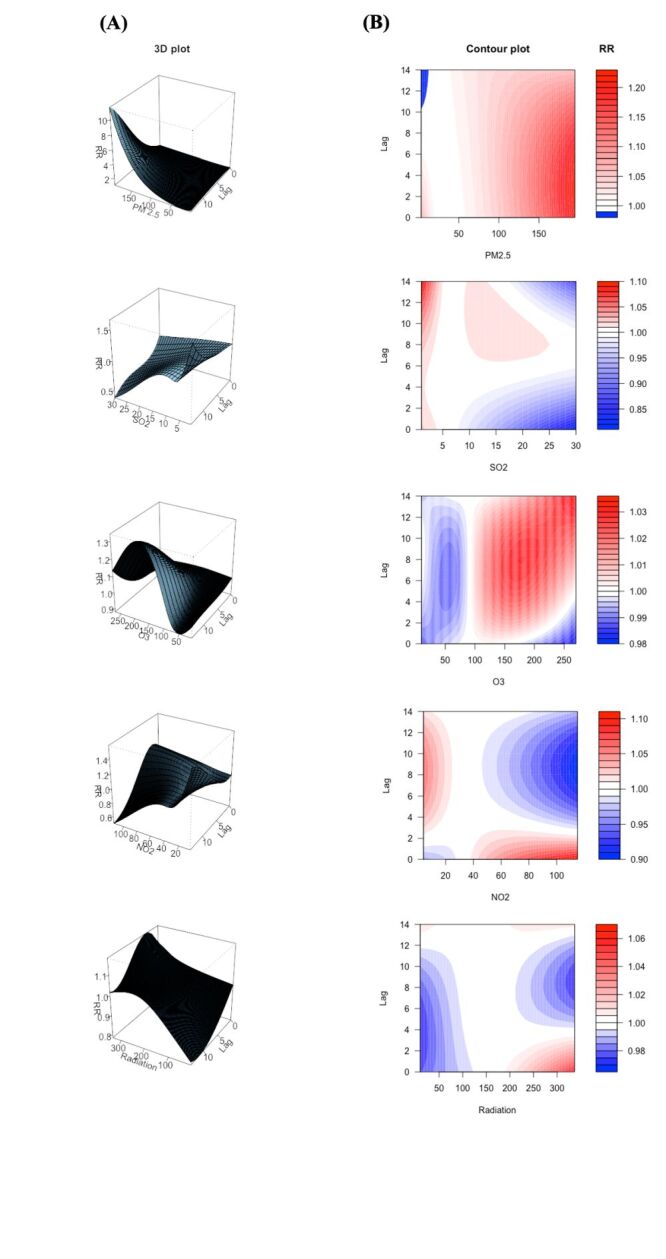
The association of pollutants and solar radiation in the relative risk of outpatient visits for pterygium. **Panel A.** The 3D illustration and **Panel B.** The contour plot showing the exposure-lag-response relationship between risk factors and pterygium visits at various lag days. Blue areas: relative risks (RR)<1; red areas: relative risks (RR)>1. CO – carbon monoxide, μm – micrometre, NO_2_ – nitrogen dioxide, O_3_ – ozone, PM_2.5_ – particulate matter less than 2.5 μm, PM_10_ – particulate matter less than 10 μm, SO_2_ – sulphur dioxide.

**Figure 2** showed the cumulative exposure-lag effect of lag days and meteorological factors on the number of outpatient visits for pterygium during the 14-day lag period. The reference value is determined by the median of each meteorological variable, of which the relative risk (RR) was defined as 1. The risk of hospitalisation seems to increase with increasing concentration of PM_2.5_, reaching the peak at maximum PM_2.5_ concentration with a lag time of 13 days (RR = 3.59; 95% CI = 1.25–10.30). The association between other pollutants and hospitalisation was less significant. O_3_ was associated with an increased risk of visits at a range from 92 to 195 ug/m^3^, with a peak RR value of 1.27 (95% CI = 1.03–1.56). Some surprising trends appeared in SO_2_ and NO_2_, with higher concentrations corresponding to lower RR values, but the 95% CI seemed to indicate that these trends were not statistically significant. As for the solar radiation, increased level of solar radiation corresponds to a gradual increase in the risk of outpatient visits, with the highest RR values reached at the max intensity and a lag of four days (RR = 1.18; 95% CI = 1.02–1.36).

**Figure 3:**
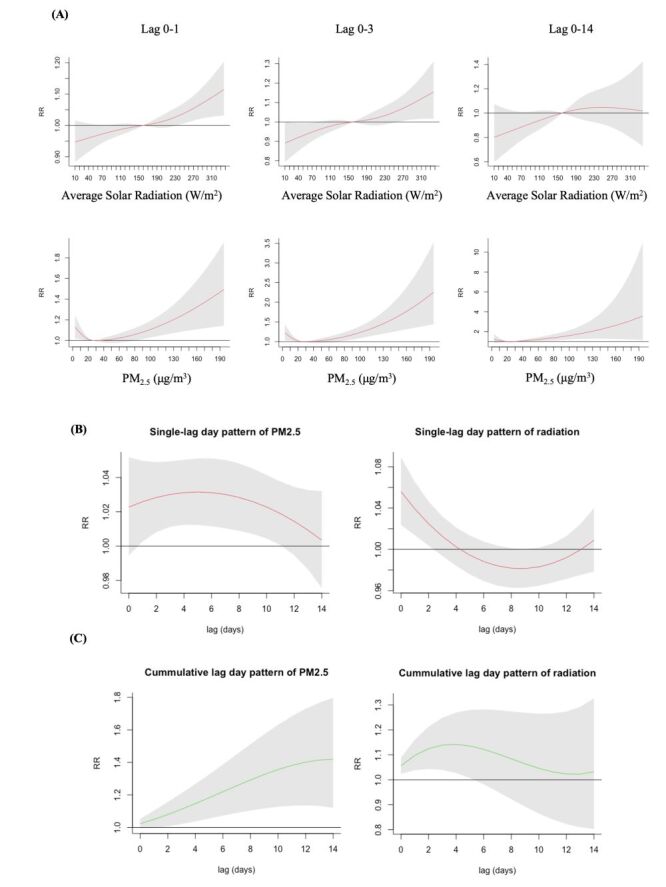
The cumulative and single-day lag effects of different solar radiation and PM_2.5_ level. **Panel A.** Overall cumulative lag effect on pterygium visits in PM_2.5_ and solar radiation at lag 0-1, lag 0-3, and lag 0-14. **Panel B.** The single-day lag effects of extremely high PM_2.5_ and solar radiation. **Panel C.** The cumulative lag effects of extremely high PM_2.5_ and solar radiation. Grey areas: 95% confidence interval (CI). μm – micrometre, μg – microgram, PM2.5 – particu­late matter less than 2.5 μm.

From the above results, we noticed that high levels of PM_2.5_ and solar radiation may be correlated with more outpatient visits. Slices at different lag-day showed that as these two meteorological variables increased, the RR values of pterygium visits tended to be higher (**Figure 3**, Panel A). Taking the 97.5 percentile as the extremely high value, the single-day lag effect and cumulative lag effect of extremely high PM_2.5_ and solar radiation were shown in **Figure 3**, Panels B–C. These results suggest that an exposure to high-level PM_2.5_ is related to increased risk of pterygium visits, based on a cumulative lag effect model of lag 0-2 (RR = 1.08; 95% CI = 1.01–1.16) to 0–14 (RR = 1.42; 95% CI = 1.12–1.80). The effect of solar radiation, on the other hand, was mainly manifested within lag 0-0 (RR = 1.06; 95% CI = 1.02–1.09) to 0-5 (RR = 1.14; 95% CI = 1.01–1.28) days.

### Subgroup analysis

We performed a subgroup analysis based on the gender and age of patients to identify sensitive subgroups for climate factors. With selecting the 2.5th percentile (P_2.5_) and the 97.5th percentile (P_97.5_) as extreme low and high levels of climate variables, **Table 2** showed the lag effect of extreme PM_2.5_ concentration (P_2.5_ = 8 μg/m^3^, P_97.5_ = 84 μg/m^3^) on people of different ages and genders under different lag times. We found that women and people aged 55–64 were more sensitive to high concentrations of PM_2.5_. In both subgroups, RR values for high PM_2.5_ levels were statistically significant between lag0-1 and lag0-14. At a lag of 14 days, the RR value of extremely high PM_2.5_ was 1.84 (95% CI = 1.30–2.59) for women *vs*. 1.56 (95% CI = 0.95–2.57) for men. At the same lag time and concentration of PM_2.5_, the RR value for people aged 55–64 was 2.10 (95% CI = 1.31–3.37), while the RR value for people under 55 was 1.20 (95% CI = 0.73–2.00) and for people over 64 was 2.05 (95% CI = 1.24–3.40).

**Table 2:** The cumulative relative risk of pterygium at extremely low and high PM_2.5_ over different lag days stratified by patients’ age and gender

Subgroups	PM_2.5_ level	lag 0-1	lag 0-3	lag 0-7	lag 0-14
Total	Extremely low	1.066 (1.018–1.115)*	1.117 (1.036–1.205)*	1.172 (1.039–1.323)*	1.109 (0.908–1.353)
	Extremely high	1.053 (1.000–1.109)*	1.118 (1.025–1.221)*	1.270 (1.097–1.470)*	1.440 (1.131–1.844)*
Age (in years)					
*Age ≥65*	Extremely low	1.055 (0.956–1.164)	1.089 (0.924–1.283)	1.095 (0.840–1.428)	0.969 (0.629–1.495)
	Extremely high	1.033 (0.926–1.152)	1.108 (0.922–1.331)	1.373 (1.012–1.862)*	2.052 (1.238–3.401)*
*55≤age <65*	Extremely low	1.006 (0.916–1.107)	1.017 (0.868–1.192)	1.042 (0.807–1.345)	1.048 (0.691–1.587)
	Extremely high	1.165 (1.051–1.290)*	1.299 (1.094–1.542)*	1.503 (1.130–2.001)*	2.101 (1.309-3.373)*
*Age <55*	Extremely low	1.159 (1.047–1.284)*	1.275 (1.075–1.511)*	1.370 (1.041–1.804)*	1.318 (0.840–2.068)
	Extremely high	0.996 (0.893–1.111)	1.027 (0.855–1.234)	1.145 (0.844–1.554)	1.203 (0.725–1.995)
Gender					
*Male*	Extremely low	1.103 (0.999–1.218)	1.181 (1.002-1.392)*	1.255 (0.962–1.637)	1.183 (0.795–1.832)
	Extremely high	1.049 (0.943–1.168)	1.136 (0.949–1.360)	1.378 (1.020–1.859)*	1.565 (0.953–2.568)
*Female*	Extremely low	1.057 (0.987–1.132)	1.095 (0.978–1.228)	1.124 (0.936–1.351)	1.077 (0.800–1.452)
	Extremely high	1.080 (1.002–1.164)*	1.157 (1.021–1.311)*	1.321 (1.073–1.628)*	1.835 (1.301–2.591)*

μm – micrometre, PM_2.5_ – particulate matter less than 2.5 µm

*P < 0.05

Similarly, **Table 3** showed the lag effects of extreme solar radiation intensity (P_2.5_ = 24 W/m^2^; P_97.5_ = 308 W/m^2^) on different subgroups We found that the subgroup effect of solar radiation on pterygium visits was less significant than that of PM_2.5_. In general, high level of sunlight was associated with an increased risk of hospitalisation in male and people over 64 years old, while low level of sunlight showed protective effects in people under 55 years old.

**Table 3:** The cumulative relative risk of pterygium at extremely low and high solar radiation over different lag days stratified by patients’ age and gender

Subgroups	Solar radiation level	lag 0-1	lag 0-3	lag 0-7	lag 0-14
Total	Extremely low	0.957 (0.912–1.004)	0.917 (0.846–0.995) *	0.855 (0.749–0.975)*	0.833 (0.668–1.040)
	Extremely high	1.105 (1.040–1.176)*	1.151 (1.040–1.274)*	1.112 (0.945–1.308)	1.028 (0.780–1.354)
Age (in years)					
*Age ≥65*	Extremely low	1.008 (0.920-1.106)	1.008 (0.865–1.175)	1.014 (0.792–1.299)	1.255 (0.826–1.906)
	Extremely high	1.125 (1.007–1.257)*	1.168 (0.971–1.404)	1.097 (0.814–1.479)	1.093 (0.663–1.802)
*55≤age <65*	Extremely low	0.945 (0.867–1.030)	0.892 (0.773–1.030)	0.800 (0.633–1.010)	0.701 (0.473–1.040)
	Extremely high	1.105 (0.995–1.227)	1.153 (0.969–1.372)	1.106 (0.835–1.464)	0.855 (0.535–1.368)
*Age <55*	Extremely low	0.919 (0.838–1.008)	0.844 (0.724-0.983)*	0.715 (0.557–0.918)*	0.580 (0.380–0.887) *
	Extremely high	1.053 (0.942–1.177)	1.056 (0.877–1.270)	0.982 (0.727–1.327)	0.966 (0.583–1.601)
Gender					
*Male*	Extremely low	0.999 (0.912–1.094)	0.942 (0.811–1.095)	0.768 (0.602–0.980)*	0.639 (0.423–0.966) *
	Extremely high	1.158 (1.037–1.292)*	1.233 (1.028–1.479)*	1.196 (0.890–1.606)	1.126 (0.686–1.847)
*Female*	Extremely low	0.932 (0.876–0.993)*	0.892 (0.804-0.990)*	0.865 (0.730–1.025)	0.885 (0.664–1.179)
	Extremely high	1.082 (1.002–1.167)*	1.105 (0.974–1.254)	1.038 (0.847–1.271)	0.923 (0.657–1.296)

*P < 0.05.

### Sensitivity assessment

For sensitivity assessment, we adjusted the df values of time (df = 7–9) and meteorological factor (df = 3–5) (Figure S1 in **the** **Online Supplementary Document**). It is observed that changing the df value does not cause significant changes in the results, indicating that the model we constructed is reliable.

## DISCUSSION

With the extensive progress of global urbanisation, it is of great significance to pay attention to whether air pollution and climate change caused by industrial development and urban traffic will aggravate ocular surface diseases in maintaining visual quality. This study is the first to conduct a lag-response analysis combining air pollutants, solar radiation and pterygium outpatient visits, indicating that in addition to those recognised long-term factors, a short-term exposure to high-level PM_2.5_ and solar radiation may also contribute to the development of pterygium, and is more significant in female and the 55–64 aged population.

According to the State of Global Air 2020, PM_2.5_ has been the largest contributor to the burden of disease worldwide caused by air pollution [19]. Due to its diameter equal to or less than 2.5 microns, PM_2.5_ can easily enter human body to produce cytotoxic effects [20]. Clinical studies in ophthalmology have shown that exposure to fine particulate matter can promote the development and exacerbation of ocular surface diseases such as dry eye, blepharitis, and allergic conjunctivitis [20–22], and even further lead to high intraocular pressure (IOP) or retinal pigment epithelial cell damage by activating the pyroptosis of cells located in the posterior segment of eyes [23–25]. Our study is the first to highlight that short-term exposure to PM_2.5_ has a strong cumulative lag effect on pterygium visits. In a time-stratified case-cross analysis based on data from ophthalmology outpatient clinics in Hangzhou, China, Fu.et al found that outpatient visits to pterygium were positively correlated with outdoor PM levels, and female patients under the age of 65 were more sensitive to PM_2.5_ than male patients, which was consistent with our results [26]. The different sensitivities to PM_2.5_ in subgroups may be caused by different lifestyles, for example, patients under the age of 65 may prefer outdoor activities than those older and therefore have more opportunities to be exposed to pollutants [27]. In addition, several studies reported that due to the lack of androgens, which have shown anti-inflammatory effects on the ocular surface, females are more sensitive to the disruption of ocular surface homeostasis than males, providing a possible biological explanation for the gender difference we found [28].

The association between PM_2.5_ and pterygium is supposed to be mediated by the limbal stem cell defect (LSCD) caused by PM_2.5_, as Hao et al. found in rat models that exposure to PM_2.5_ for weeks can lead to depletion of limbal stem/progenitor cells (LSPCs) and can affect the nutrient supply and self-renewal of LSPCS by disrupting the limbal microenvironment [29]. Limbal stem cells are located at the junction area of the cornea and conjunctiva, being responsible for maintaining the integrity of the corneal surface and the continuous renewal of the corneal epithelium, and inhibiting the invasion of conjunctiva epithelial cells into the transparent cornea through proliferation pressure [30]. Focal limbal injury has been observed leading to fibrovascular invasion and conjunctivalisation of the cornea, which is the initial state of pterygium [31]. Correspondingly, several clinical studies have found that corneal limbal stem cell transplantation (LSCT) after the excision of pterygium can reduced the risk of pterygium recurrence, providing support for this hypothesis from another aspect [32].

Our study also supports the role of solar radiation in the pathogenesis of pterygium. UV rays in sunlight are recognised as one of the most important risk factors of pterygium, which affect the proliferation, apoptosis and migration of limbal and conjunctival cells through activation of biochemical pathways such as ERK-MAPK and NF-κβ, as well as direct damage on genetic material [2,33]. Although it has been found that short-term UV irradiation contributes to the formation of local anti-inflammatory microenvironment, the reduced differentiation function of limbal stem cells and local microcirculation disturbance induced by UV exposure still pose a threat to the physiological homeostasis of limbal niche [34]. In addition, Igarashi et al. also found that UV irradiation can cause the up-regulation of sphingosine 1 phosphate (S1P) and its downstream Rho signaling pathway in conjunctival tissue, promote the activation of conjunctival fibroblasts, and enhance the invasiveness of pterygium [35]. In response to this risk factor, new protective measures are being developed. Notara et al. reported that a UV blocking contact lens (UVBCL) showed satisfying preventive effect in models of UVB-induced DNA damage in limbal epithelial cells and fibroblasts in vitro, and could effectively reduce the secretion of angiogenic cytokines, which may be a promising preventive measure against pterygium recurrence [36]. Supplementing nerve growth factor (NGF) and applying DNA repairing enzymes such as cyclobutane pyrimidine dimers photolyase (CPDPL) and T4 endonuclease V (T4N5) have also shown good efficacy in UV-related ocular surface damage [37–39]. We look forward to more clinical trials to further scale up these preventive or therapeutic measures.

This study still has several limitations. First, this study was a geographic study and the data collection was limited in Shanghai. Our findings may be further generalised if multicenter studies can be supported by more clinical centers from different geographic or social environments. In addition, as this is a retrospective study and the recording of cases was limited by the outpatient information system, we cannot obtain more detailed personal information of the patients such as economic level, living environment, occupational exposure, *etc*. These personal factors can also affect a patient's sensitivity to climate change and air pollution.

## CONCLUSIONS

In summary, our study found that PM_2.5_ and UV radiation levels are associated with the risk of outpatient visits for pterygium, and reported that high concentration of PM_2.5_ has a strong cumulative lag effect on pterygium visits, especially for females and the 55–64 age subgroup. These results highlight the importance of sustainable public health strategies, such as air quality regulations and UV protection campaigns. We are looking forward to more studies to validate the biological mechanisms of PM_2.5_-induced ocular damage and to access more targeted interventions in the future, such as environmental exposure guidelines for high-risk populations.

## Additional material


Online Supplementary Document
