# Supplementary material for: The nonlinear impact of air pollutants and solar radiation exposure on the risk of hospitalisation for pterygium among adults in Shanghai, China: a time series analysis
Source: J Glob Health. 2025 Apr 11;15:04110. doi: 10.7189/jogh.15.04110 (PMC11987575; doi:10.7189/jogh.15.04110)
Supplement: Online Supplementary Document [file jogh-15-04110-s001.pdf]

Supplement to: Zeng H, Tan Y, Lin T, Gong L. The nonlinear impact of air pollutants and solar radiation exposure on the risk of hospitalisation for pterygium among adults in Shanghai, China: a time series analysis. J Glob Health. 2025;15:04110.

			Warm season		Cold season	
**Variables**	**Mean**	**SD**	**Peak value**	**Median**	**Peak value**	**Median**
Daily average temperature (℃)	17.41	8.43	33.46	24.60	28.89	10.10
Relative humidity (%)	76.44	10.39	96.13	79.62	98.25	74.47
solar radiation intensity (W/m^2)	164.74	81.69	336.2	222.80	282.43	132.98
PM2.5 (ug/m^3)	31.72	20.92	100.00	23.00	191.00	29.00
PM10 (ug/m^3)	46.46	26.91	325.00	36.00	311.00	44.00
SO_2 (ug/m^3)	7.36	3.12	21.00	6.00	29.00	7.00
NO_2 (ug/m^3)	36.48	17.02	81.00	28.00	115.00	40.00
CO (mg/m^3)	0.65	0.19	1.20	0.60	1.80	0.65
O_3 (ug/m^3)	97.84	39.67	269.00	113.00	186.00	78.00

Table S1 Descriptive summary of daily meteorological factors and air pollutants in Shanghai, 2017-2023.

CO – carbon monoxide, NO_2 – nitrogen dioxide, O_3 – ozone, PM2.5 – particulate matter less than 2.5 μm, PM10 – particulate matter less than 10 μm, SO_2 – sulphur dioxide, SD – standard deviation

	**Temperature**	**Relative Humidity**	**PM2.5**	**PM10**	**O3**	**SO2**	**NO2**	**CO**	**Solar Radiation**
Temperature	1.00	0.35	-0.32	-0.27	0.49	-0.28	-0.41	-0.32	0.43
Relative Humidity		1.00	-0.12	-0.41	-0.17	-0.37	-0.14	-0.07	-0.41
PM2.5			1.00	**0.72**	0.09	0.57	0.68	**0.80**	0.02
PM10				1.00	0.16	0.55	0.54	0.53	0.19
O3					1.00	0.08	-0.18	-0.05	0.67
SO2						1.00	0.55	0.46	0.13
NO2							1.00	0.65	-0.16
CO								1.00	-0.14
Solar Radiation									1.00

Table S2. Spearman's correlation coefficients of daily meteorological factors and air pollutants in Shanghai, 2017-2023

CO – carbon monoxide, NO_2 – nitrogen dioxide, O_3 – ozone, PM2.5 – particulate matter less than 2.5 µm, PM10 – particulate matter less than 10 µm, SO_2 – sulphur dioxide

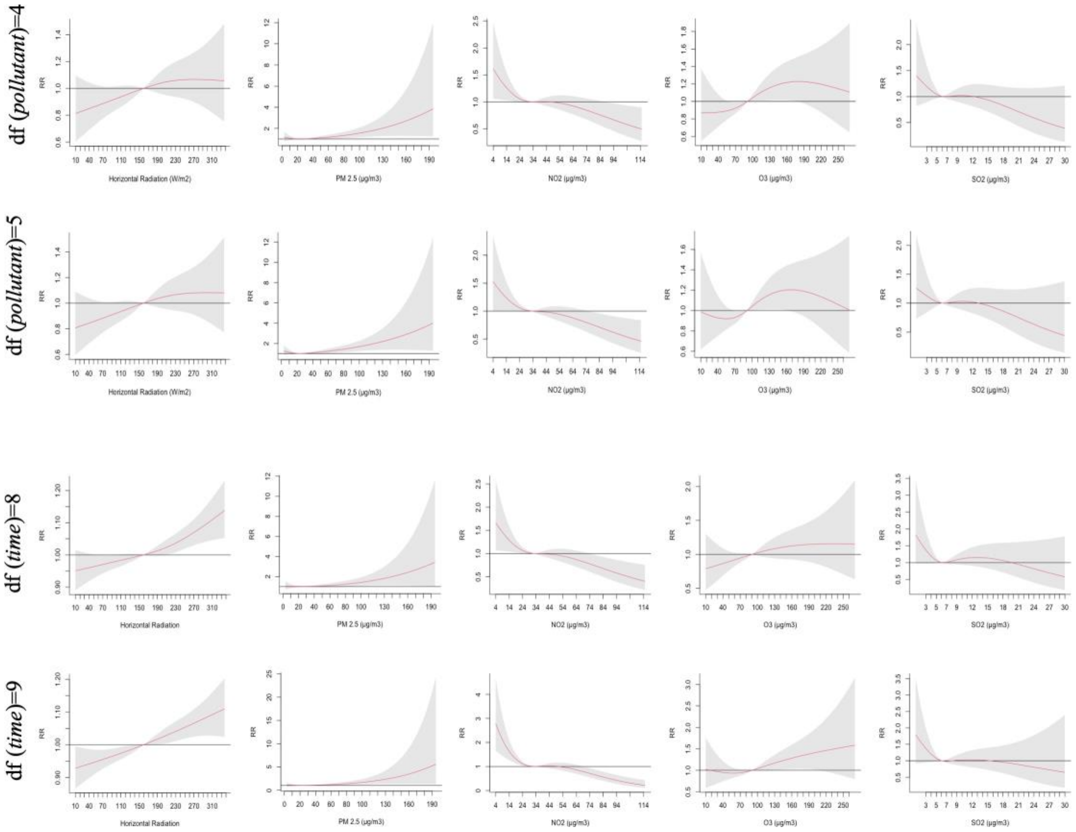

Figure S1. Sensitivity assessments of model by adjusting the df values.
CO – carbon monoxide, NO_2 – nitrogen dioxide, O_3 – ozone, PM2.5 – particulate matter less than 2.5 µm, SO_2 – sulphur dioxide
